# Bioceramic micro-fillers reinforce antibiofilm and remineralization properties of clear aligner attachment materials

**DOI:** 10.3389/fbioe.2023.1346959

**Published:** 2024-01-09

**Authors:** Wenhui Jiang, Zhejun Wang, Yinghong Zhou, Ya Shen, Edwin Yen, Bingshuang Zou

**Affiliations:** ^1^ Division of Orthodontics, Department of Oral Health Science, Faculty of Dentistry, The University of British Columbia, Vancouver, BC, Canada; ^2^ Division of Endodontics, Department of Oral Biological and Medical Sciences, Faculty of Dentistry, The University of British Columbia, Vancouver, BC, Canada; ^3^ School of Dentistry, The University of Queensland, Brisbane, QLD, Australia

**Keywords:** bioactive glass, bioceramic filler, bredigite, clear aligner attachment, plaque biofilm

## Abstract

**Introduction:** Clear aligners, while offering a more hygienic alternative to fixed appliances, are still associated with challenges including plaque accumulation and enamel demineralization. The aim of the present study was to investigate the antibiofilm and remineralization effectiveness of innovative flowable composite attachments containing bioceramic micro-fillers.

**Methods:** Four experimental attachments were formulated and bonded to human enamel specimens: 3M Filtek Supreme flowable composite (Filtek SF) + 10% bioactive glass 45S5 (BAG), Filtek SF + 30% BAG, Filtek SF + 10% Bredigite (BRT), Filtek SF + 30% BRT. Plaque biofilms were grown on the bonded enamel using a standardized protocol and the biofilm-killing effect was assessed by confocal laser scanning microscopy and scanning electron microscopy. Vickers microhardness was measured to evaluate the remineralization effect of the attachments containing bioceramic fillers after acid challenge. Shear bond test was performed to assess the bonding strength.

**Results:** Attachments with bioceramic fillers significantly inhibited plaque biofilm growth in 3 weeks on enamel, contributing over 20% bacterial cell killing in 10% filler groups and over 30% killing in 30% filler groups. All four experimental groups demonstrated significantly higher microhardness values than the control group without fillers on the attachment side. The shear bonding strength was not compromised in the attachments with micro-fillers.

**Discussion:** Proper incorporation of bioceramic micro-fillers in attachments provides an innovative approach for clear aligner therapy with reinforced antibiofilm and remineralization effects without weakening shear bonding strength.

## 1 Introduction

Over the past few decades, there has been a growing demand for alternatives to conventional fixed orthodontic appliances, driving the development of more comfortable and aesthetically pleasing appliances. This shift has led to the emergence of clear aligner therapy (Khosravi et al., 2017). Removable thermoplastic clear aligners, worn sequentially by patients, have gained popularity for achieving targeted orthodontic results (Weir, 2017). However, it’s worth noting that composite clear aligner attachments tend to exhibit increased biofilm accumulation compared to other restorative materials (Bourbia et al., 2013; Bichu et al., 2023).

Despite claims of a more hygienic design and expectations of fewer white spot lesions with clear aligners compared to traditional fixed appliances (Buschang et al., 2019), enamel demineralization and plaque accumulation remain critical side effects, particularly with prolonged wear (Santonocito and Polizzi, 2022). A significant increase in the total bacterial load with both types of appliances was reported (Mummolo et al., 2020). A recent randomized clinical trial has reported a significantly larger lesion area in the clear aligner group at T1 (3 months after treatment) than at T0 (beginning of treatment) and the fixed appliance group (Albhaisi et al., 2020). This increase in white spot lesions during clear aligner therapy may be attributed to limitations in saliva flow, natural buffering, and remineralizing properties (Azeem and Hamid, 2017). Resin composites, known to enhance bacterial growth (Beyth et al., 2008), contribute to a high cariogenic challenge due to bacterial biofilm accumulation around attachments during intraoral wear.

Previous antibiofilm studies have predominantly focused on fixed orthodontic appliances rather than clear aligner attachments (Badawi et al., 2003; Zhang et al., 2015a; Ferreira et al., 2019), often employing single-species bacteria (e.g., *Streptococcus mutans* and *Lactobacilli*) rather than the multispecies bacteria commonly found in white spot lesions (Chin et al., 2006; Zhang et al., 2015b). However, bacteria in white spot lesions all originate from multispecies plaque on the tooth surface (Cheng et al., 2012; Beerens et al., 2017). Most commercially available composite resins exhibit minimal antibiofilm effects, with biofilms tending to accumulate on them over time (Beyth et al., 2007; Khalichi et al., 2009). Additionally, these composites can be degraded by enzymes from saliva and bacteria (Montoya et al., 2021). The acid produced by bacterial biofilms can cause enamel demineralization around the clear aligner attachments (Zhang et al., 2017). Therefore, exploring novel bioactive attachment materials that enhance antibiofilm and remineralization properties without compromising bonding strength is essential in clear aligner therapy.

Existing antibacterial/antibiofilm agents containing leachable compounds (e.g., chlorhexidine), antibiofilm peptides, and filler nanoparticles (e.g., zinc and silver) have major limitations of short-term effectiveness and an inability to regenerate mineral content lost due to hard enamel damage (Stewart and Finer, 2019; Makvandi et al., 2020). Bioactive glass (BAG) has been identified for its ability to inhibit enamel demineralization in orthodontic bonding resins (Lee et al., 2018). Two studies have highlighted the robust antimicrobial activity of bioactive glass S53P4 against *S. aureus* and *Pseudomonas aeruginosa* (Coraça-Huber et al., 2014; Drago et al., 2014). Bredigite (BRT) bioceramics have demonstrated osteogenic effects on dentin and plaque growth suppression (Shen et al., 2016). However, the effects of using BAG and BRT as micro-fillers in clear aligner attachments remain relatively unexplored.

Achieving long-term clinical success necessitates an ideal material that is multifunctional, capable of overcoming limitations of individual formulations. Despite the potential offered by bioactive fillers, there is a lack of standardized protocols to systematically assess antibiofilm, remineralization, and bonding in one study, specifically mirroring the clinical situation of clear aligner therapy. Existing *in vitro* studies have often focused on evaluating the antimicrobial effect of bonding materials independently on composite resin disks (Zhang et al., 2015a; Zhang et al., 2015b; Altmann et al., 2017) rather than on attachment bonded enamel. Thus, the development of a standardized model is crucial to simulate the clinical reality of clear aligner therapy.

This project aimed to formulate two bioceramic micro-filler-based attachments and systematically investigate their antibiofilm and remineralization effects, as well as assess shear bond strength on a standardized platform, providing a comprehensive evaluation of their performance in a manner that closely replicates the clinical conditions of clear aligner therapy.

## 2 Materials and methods

### 2.1 Enamel sample and attachment mould preparation

This study was approved by the Clinical Research Ethics Committee of the University of British Columbia (certificate H22-01985). Sixty caries-free human premolars subjected to orthodontic extraction were collected. The extracted teeth were stored in deionized water at 4°C. The crown of each premolar was sectioned perpendicularly to the long axis of the tooth at the cementoenamel junction using a low-speed water-cooled diamond saw (Isomet, Buehler, Lake Bluff, IL, United States).

A platinum-catalyzed silicone rubber (Ecoflex™ Smooth-on, Macungie, PA, United States) was used to fabricate flexible silicone moulds used for the preparation of attachments. Each mould featured an inner diameter of 2 mm (length) × 4 mm (width) × 3 mm (thickness) and an outer diameter of 4 mm (length) × 6 mm (width) × 3 mm (thickness), following the manufacturer’s guidelines.

**FIGURE 1:**
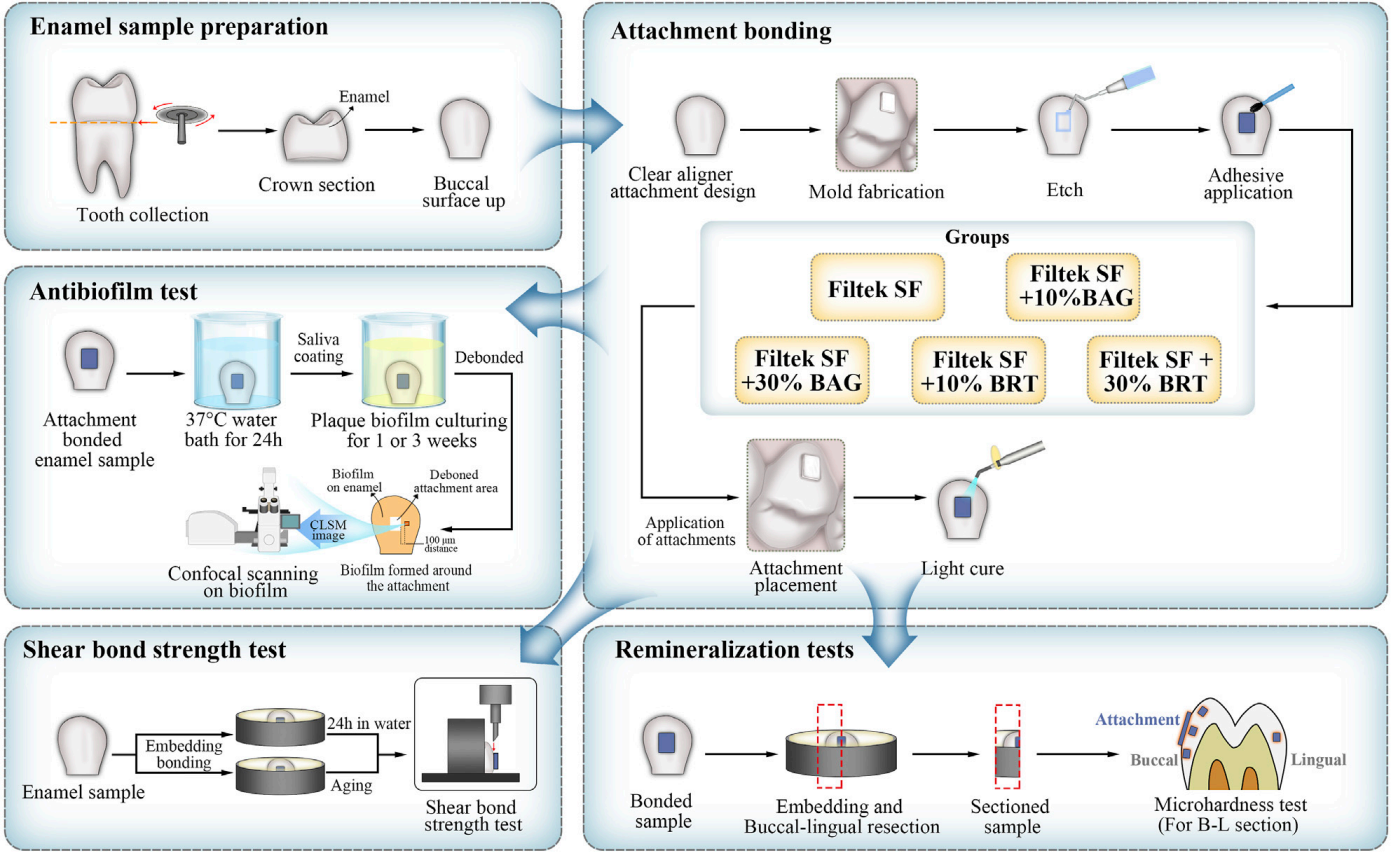
Schematic illustration of the experimental design in the present study.

The experimental design is outlined in the schematic diagram presented in Figure 1.

### 2.2 Bioceramic fillers and attachment material preparation

The composition of bioactive glass 45S5 (BAG), bredigite (BRT), and the flowable composite attachment material (Filtek SF) is listed in Table 1. Bioactive glass 45S5 contains 45 wt% SiO_2_, 24.5 wt% CaO, 24.6 wt% Na_2_O, and 5.8 wt% P_2_O_5._ According to a previously described protocol, BRT powders were synthesized by a sol-gel method using raw materials including (C_2_H_5_O)_4_Si, Mg(NO_3_)_2_·6H_2_O and Ca(NO_3_)_2_·4H_2_O (Sigma Aldrich, St Louis, MO, United States) (Wu and Chang, 2007).

**TABLE 1:** Composition of the bioceramic micro-fillers and their incorporated flowable composite attachments.

Materials	Manufacturer	Components
Bioactive glass 45S5 (BAG)	OSspray Ltd., London, United Kingdom	SiO_2_, CaO, Na_2_O, P_2_O_5_
Bredigite (BRT)	—	(C_2_H_5_O)_4_Si, Mg(NO_3_)_2_·6H_2_O, Ca(NO_3_)_2_·4H_2_O
3M Filtek Supreme Flowable (Filtek SF)	3M ESPE, St Paul, MN, United States	BIS-GMA, TEGDMA, Si, Zr, YbF_3_

BIS-GMA, Bisphenol A dimethacrylate; TEGDMA, triethyleneglycol dimethacrylate.

Flowable composite attachments containing four different types of experimental bioceramic micro-filler were formulated freshly before the attachment bonding. The flowable composite attachment, 3M Filtek Supreme Flowable (Filtek SF), was manually blended with 10 wt% BAG, 30 wt% BAG, 10 wt% BRT and 30 wt% BRT powders respectively using a metallic spatula. This mixture was further homogenized in an amalgamator (Ivoclar Vivadent, Mississauga, ON, Canada) for 30 s. Subsequently, the mixture was dispensed into a resin dispenser and injected into the attachment molds.

### 2.3 Enamel-attachment bonding

The 60 teeth were randomly divided into 5 groups, each consisting of 12 teeth. The groups were defined as follows: 3M Filtek Supreme Flowable (Filtek SF) only (control group), Filtek SF + 10% BAG, Filtek SF + 30% BAG, Filtek SF + 10% BRT, and Filtek SF + 30% BRT.

The buccal enamel surface of each tooth was etched with 35% phosphoric acid (Pulpdent, Watertown, MA, United States) for 30 s, followed by a thorough 30-s water rinse and complete drying with oil-and-moisture-free air. A thin uniform layer of Assure universal bond (Reliance orthodontic products, Itasca, IL, United States) was applied to the etched surfaces and cured for 10 s. Subsequently, a silicone mold, as described in Section 2.1 was gently positioned on the center of the buccal enamel surface and pressed firmly into place. The various attachment materials, corresponding to the five groups, were dispensed into the rubber mold.

Excess adhesive around the attachment was meticulously removed using a cotton pellet, ensuring the integrity of the mold. The attachment underwent light-curing (LED-B, Woodpeck Inc., Guilin, China) for 30 s with the mold in place. Following mold removal, an additional 20 s of light-curing was administered. All attachment-bonded samples were then immersed in deionized water at 37°C for 24 h before further utilization.

### 2.4 Biofilm model

Supragingival plaque samples were obtained from the first or second premolars of two adult volunteers: one non-clear aligner wearer (donor 1) and another wearing clear aligner for 3 months (donor 2). Collected plaques from both donors were separately mixed in brain–heart infusion broth (BHI) (Becton Dickinson, Sparks, MD) by pipetting. Bacterial suspensions from each donor were standardized to an optical density (OD) of 0.10 (150 μL at 405 nm) using a microplate reader (Model 3350; Bio-Rad Laboratories, Richmond, CA, United States).

In an effort to replicate clinical conditions, enamel samples with attached attachments were coated with saliva. The saliva was collected from each volunteer (at least 1.5 h after meal) in sterile 14-mL polypropylene tubes (Corning, NY, United States) and filtrated using sterilized 0.2 μm syringe filters. Each bonded enamel surface was coated with 500 μL infiltrated saliva for 4 h in a well of a sterile 24-well polystyrene cell culture plate (Corning, NY, United States) before initiating biofilm culturing.

Subsequently, the saliva-coated enamel samples were placed in the wells of a 24-well cell culture plate, each containing 1.8 mL of BHI. Each well was inoculated with 0.2 mL of dispersed plaque suspension from either donor 1 or donor 2. The samples were incubated in the BHI-plaque suspension in air at 37°C for 1 and 3 weeks. For each time point in each group and from each donor, three attachment-bonded samples were utilized. Fresh BHI broth was replaced weekly for the 3-week specimens.

### 2.5 Evaluation of the antibiofilm effect

The plaque biofilm on the enamel around attachments was subjected to bacterial viability staining and observed under a confocal laser scanning electron microscopy (CLSM). Attachment-bonded samples cultured with biofilm for 1 and 3 weeks were rinsed in 0.85% physiological saline for 1 min. Subsequently, the attachments on the enamel samples were meticulously removed using debonding pliers (HuFriedy Group, Chicago, IL, United States).

LIVE/DEAD BacLight Bacterial Viability kit L-7012 (Molecular Probes, Eugene, OR, United States), containing a two-component dye (SYTO 9 and propidium iodide in a 1: 1 mixture) in a solution, was used for staining the biofilm following the manufacturer’s protocols. The excitation/emission maxima for these dyes were 480/500 nm for the SYTO 9 whole cell stain and 490/635 nm for the dead cell stain propidium iodide. A confocal laser scanning electron microscopy (FV10i-LIV, Olympus, Tokyo, Japan) was used to analyze the plaque biofilm fluorescence. Fluorescence from each stained cell was viewed using a CLSM at a 512 × 512 pixel scan area with a 10 × lens.

For each subgroup, five randomly selected biofilm areas at a distance of 100 μm away from the debonded attachment and biofilm interface were scanned by CLSM. The biofilms were scanned with a 1.5 μm step size from top to bottom, resulting in 15 scanned areas (n = 15) for each subgroup. The confocal images were analyzed and quantitated (live/dead ratios) using the Imaris 7.2 software (Bitplane Inc., St Paul, MN, United States). The total biovolume of the biofilm in the scanned area was calculated, and the volume ratio of red fluorescence to green and red fluorescence indicated the proportion of killed plaque bacteria.

The morphology of plaque biofilms cultivated around various attachments after a 3-week culturing period was investigated through scanning electron microscopy (SEM). An additional three samples from each group with plaque biofilm growth were prepared following the biofilm sample preparation procedure described above. The samples for SEM were prefixed with phosphate-buffered 2.5% glutaraldehyde for 10 min and then immersed in 2 mL 1% osmium tetroxide for 1 h. Subsequently, the specimens were dehydrated through a series of increasing ethanol concentrations (50%, 70%, 80%, and 100%). The dehydrated samples were dried by using a critical point drier (Samdri-795; Tousimis Research Corporation, Rockville, MD), sputter-coated with iridium (Leica EM MED020 Coating System, Tokyo, Japan), and examined by SEM (Helios Nanolab 650, FEI, Eindhoven, the Netherlands) at an accelerating voltage of 3 kV.

### 2.6 Remineralization test

An additional set of 30 enamel samples (6 for each attachment-bonded group) were prepared as outlined in Section 2.3 for microhardness evaluation.

Demineralization was conducted using 0.1 M lactic acid, with the pH adjusted to 4.0 (Zhang et al., 2018). Each attachment-bonded enamel sample was placed flat in a well of a 12-well plate (Corning, NY, United States) with the buccal surface in contact with the bottom of the well. The attachment bonded interface was fully immersed in 3 mL of lactic acid solution at 37°C for 28 days (Liu et al., 2018) and the lingual enamel surface was exposed. The demineralization solution was refreshed on a weekly basis.

Each attachment-bonded tooth was hemi-sectioned sagittally through the center of the attachment into mesial and distal halves (12 tooth-halves; n = 12) using a low-speed diamond saw (Isomet, Buehler, Lake Bluff, IL, United States) under water cooling. The sectioned specimens were embedded in self-curing epoxy-resin (Epo Thin 2, Buehler, Lake Bluff, IL, United States), leaving the sectioning side exposed. The resin-embedded specimens were wet polished using 600-grit, 1000-grit and 1200-grit silicon carbide grinding papers (CarbiMet; Buehler Ltd., Lake Bluff, IL, United States) under constant water irrigation. Final polishing was achieved with 1 μm diameter diamond cream and polishing-cloth disk (Buehler, Lake Bluff, IL, United States).

The Vickers microhardness of each specimen was measured using a microhardness tester (Micromet 3 microhardness tester; Buehler Ltd., United States) equipped with a Vickers diamond indenter at a load of 200 g and a dwell time of 15 s. The Vickers hardness (HV) was calculated using the formula: HV = 1.8549 (F/d^2^), where F is the load and d is the mean of the two diagonals produced by the indenter (Nekoofar et al., 2010).

Four indentations were made in each embedded tooth-half from four different locations. On the buccal surfaces, three indentations were made under the attachment, at the coronal and cervical edges of the attachment, respectively. An additional indentation was made on the lingual enamel surface. All four indentations were made at 60 μm from the external enamel surface. In total, 12 indentations were made for each position per group (n = 12). The mean and standard deviation of microhardness values for each group were calculated.

### 2.7 Shear bond testing

Thirty enamel samples were prepared as described in Section 2.1 with six samples for each group. The shear bond test was performed according to a previously published protocol (Hong et al., 2022). Teeth samples were mounted in an acrylic mold (Ultradent Products, South Jordan, UT, United States) using self-curing resin (Epo Thin 2, Buehler, Lake Bluff, IL, United States), positioning the buccal surface for exposure. Specimens (height = 2.38 mm, diameter = 2.00 mm) of flowable composites from different groups were fabricated using a bonding clamp and bonding mold inserts (Ultradent Products, South Jordan, UT, United States). Enamel surfaces were etched and bonded with different flowable composites from the five groups using the same bonding protocol outlined in Section 2.3. Each group was equally divided into two subgroups. The first subgroup samples were immersed in deionized water for 24 h before shear bond testing. Samples from the second subgroup were aged in 0.1 M lactic acid (pH = 4) for 28 days before shear bond testing.

Individual specimens were secured using a metal base clamp (Ultradent Products, South Jordan, UT, United States). The buccal axis of the sample was oriented so that the labial surface was parallel to the applied force. A crosshead assembly chisel (Ultradent Products, South Jordan, UT, United States) attached to a universal Testing Machine (Instron, ElectroPuls E10000 Linear-Torsion, Shimadzu, Japan) was positioned over the upper part of the attachment and parallel to the bonded interface. An occlusogingival load was applied at a crosshead test speed of 0.5 mm/min until the attachment detached. The force required for debonding was recorded and expressed in megapascals (MPa). Shear bond strength (MPa) was calculated as the debonding force divided by the attachment surface area (mm^2^).

Following detachment, each enamel surface was examined under a stereomicroscope (Nikon Eclipse Ci, Tokyo, Japan) to analyze the failure mode. The adhesive remnant index (ARI) was determined based on the presence of remaining adhesive on enamel, classified as follows: 0 = no adhesive remaining on enamel; 1 = less than half of the adhesive remaining on enamel; 2 = more than half of the adhesive remaining on enamel; 3 = all the adhesive remaining on enamel.

### 2.8 Statistical analysis

Statistical analyses were conducted using SPSS 29 software (SPSS, Chicago, IL, United States) for Windows. The mean values with standard deviation were used to present all data derived from confocal and microhardness tests, as well as shear bond strength assessments. The homogeneity of variance was evaluated using the Levene’s test. Univariate analysis of variance (ANOVA) was applied, and *post hoc* multiple comparisons were conducted to isolate and compare the significant results at a 5% significance level. The differences in the ARI scores between groups were analyzed by the Kruskal–Wallis test and the Mann-Whitney test at a significance level of *p* < 0.05.

## 3 Results

### 3.1 Antibiofilm effect

**FIGURE 2:**
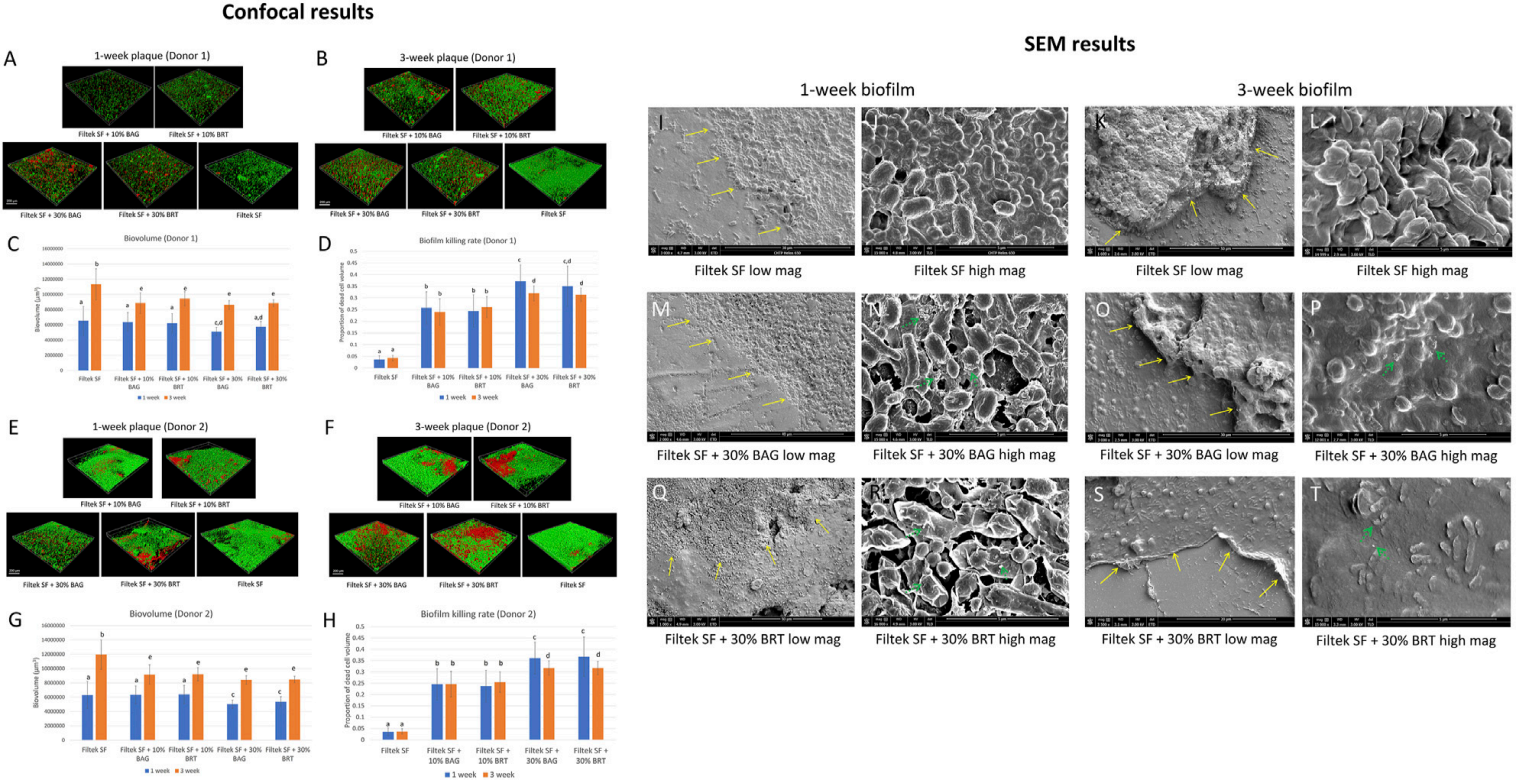
Bioceramic micro-filler containing clear aligner attachment impacts on oral plaque biofilm. **(A)** Three-dimensional constructions of CLSM scans of 1-week plaque biofilm (donor 1) formation around the attachments; **(B)** Three-dimensional constructions of CLSM scans of 3-week plaque biofilm (donor 1) formation around the attachments; **(C)** Total biovolume of plaque biofilm formed in 1 and 3 weeks from donor 1; **(D)** The proportion of the killed biofilm microbial cells of the entire plaque biofilm from donor 1. **(E)** Three-dimensional constructions of CLSM scans of 1-week plaque biofilm (donor 2) formation around the attachments; **(F)** Three-dimensional constructions of CLSM scans of 3-week plaque biofilm (donor 2) formation around the attachments; **(G)** Total biovolume of plaque biofilm formed in 1 and 3 weeks from donor 2; **(H)** The proportion of the killed biofilm microbial cells of the entire plaque biofilm from donor 2; SEM micrographs of 1-week-old biofilm exposure to Filtek SF under low **(I)** and high **(J)** magnifications. SEM micrographs of 3-week-old biofilm exposure to Filtek SF under low **(K)** and high **(L)** magnifications. SEM micrographs of 1-week-old biofilm exposure to Filtek SF + 30% BAG under low **(M)** and high **(N)** magnifications. SEM micrographs of 3-week-old biofilm exposure to Filtek SF + 30% BAG under low **(O)** and high **(P)** magnifications. SEM micrographs of 1-week-old biofilm exposure to Filtek SF + 30% BRT under low **(Q)** and high **(R)** magnifications. SEM micrographs of 3-week-old biofilm exposure to Filtek SF + 30% BRT under low **(S)** and high **(T)** magnifications. The yellow arrow shows interface of attachment and biofilm. The green arrow shows the cell lysis effect. Different letters in C, D, G and H indicate statistical differences (*p* < 0.05).

No significant difference was found in the sensitivity of biofilms (both biovolume and percentage of killed biofilm bacteria) from the two donors for attachments containing different bioceramic micro-fillers (*p* > 0.05) (Figures 2C, D, G, H).

Significant reductions in 3-week-old biofilm biovolume were observed with the addition of 10% and 30% bioceramic micro-fillers in comparison to the Filtek SF control, particularly evident in 3-week-old biofilms (Figures 2B–D, F–H). Filtek SF + 30% BAG demonstrated up to 29.7% reduction in 3-week-old biofilm biovolume compared to Filtek SF controls (Figures 2C, G; Supplementary Datasheet S1). While 1-week-old biofilm groups with 10% BAG and 10% BRT exhibited similar biovolume to Filtek SF controls, the 30% micro-filler groups displayed increased resistance to biofilm growth (Figures 2C, G).

Attachments containing either BAG or BRT demonstrated statistically significant increase in biofilm bacteria killing compared to attachment with no fillers (*p* < 0.05) (Figures 2A, B, D–F, H). The proportion of killed biofilm bacterial cells was significantly correlated with the portion of bioceramic fillers in the flowable composite attachments. Attachments containing 30% bioceramic micro-fillers were superior to groups with 10% fillers in biofilm-killing efficacy for both donors (*p* < 0.05) (Figures 2D, H). The combination of Filtek SF and 30% BAG or 30% BRT killed 35%–37% of the 1-week-old plaque bacteria for both donors (Figures 2D, H; Supplementary Datasheet S1). The addition of 10% BAG or 10% BRT into Filtek SF resulted in 24%–26% biofilm killed (Figures 2D, H; Supplementary Datasheet S1). When 30% of bioceramic fillers were added, the rate of biofilm killing was lower in the 3-week-old plaque biofilm compared to the 1-week-old biofilm except for the 30% BRT group from donor 1 (Figures 2D, H). No statistically significant difference was detected between 1-week-old biofilm and 3-week-old biofilm in 10% bioceramic micro-fillers and no-filler control groups (*p* > 0.05) (Figures 2D, H).

The multispecies composition and distribution of the plaque biofilm after debonding was validated by SEM, showing cocci, rods and filaments within the biofilms forming mixed communities along the debonded interface (Figures 2I, K, M, O, Q, S). Control biofilms growing around non-filler-containing Filtek SF demonstrated well-organized network structures with smooth surfaces and no disrupted bacterial cells (Figures 2I–K, L). In contrast, cell lysis was evident in biofilms grown around attachments containing bioceramic micro-fillers (Figures 2N, P, R, T). The bacterial cell membranes of 1-week-old biofilms became wrinkled in the 30% BRT group (Figure 2R), and both the 30% BAG and BRT groups exhibited fine particles released within the biofilm structures (Figures 2N, P, R, T).

### 3.2 Microhardness test

**FIGURE 3:**
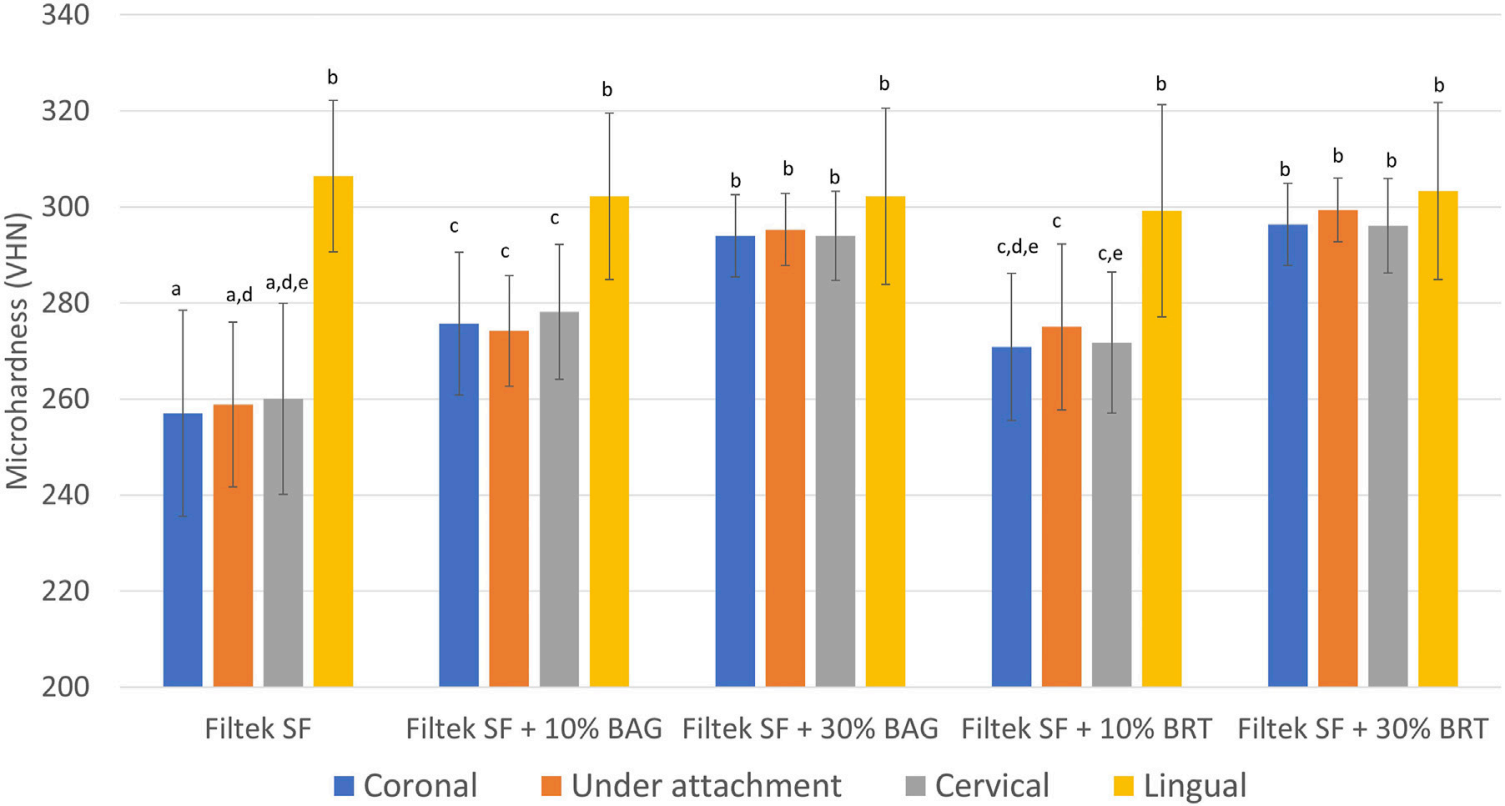
Mean surface Vickers microhardness (VHN) obtained from different locations in enamel samples bonded with Filtek SF, Filtek SF + 10% BAG, Filtek SF + 30% BAG, Filtek SF + 10% BRT, Filtek SF + 30% BRT. Columns labeled with different letters are statistically significantly different (*p* < 0.05).

Significant differences in microhardness values were found depending on the concentration of bioceramic micro-fillers in the attachments. Vickers hardness values (VHN) derived from microhardness testing were illustrated in Figure 3; Supplementary Datasheet S1. Lingual enamel surfaces that were not exposed to the demineralization solution displayed the highest microhardness values ranging between 299 and 306 (Figure 3; Supplementary Datasheet S1). In contrast, the enamel hardness on the buccal surface was significantly reduced in the Filtek SF group without the addition of bioceramic micro-fillers. No statistically significant difference in microhardness was detected among the positions of the indentation on the buccal enamel (coronal, under attachment, and cervical) for all groups (*p* > 0.05) (Figure 3).

With the inclusion of 10% bioceramic micro-fillers, the Filtek SF + 10% BAG group exhibited significantly higher microhardness values than the Filtek SF control (*p* < 0.05). The Filtek SF + 10% BRT group showed no significant difference compared to the Filtek SF + 10% BAG group (*p* > 0.05). However, groups with 10% micro-fillers still demonstrated significantly lower microhardness than the lingual control (*p* < 0.05). As the percentage of BAG and BRT reached 30% in weight, microhardness values from coronal, under attachment, and cervical enamel surfaces showed no statistically significant difference from the lingual controls (*p* > 0.05) (Figure 3).

### 3.3 Shear bond strength

**FIGURE 4:**
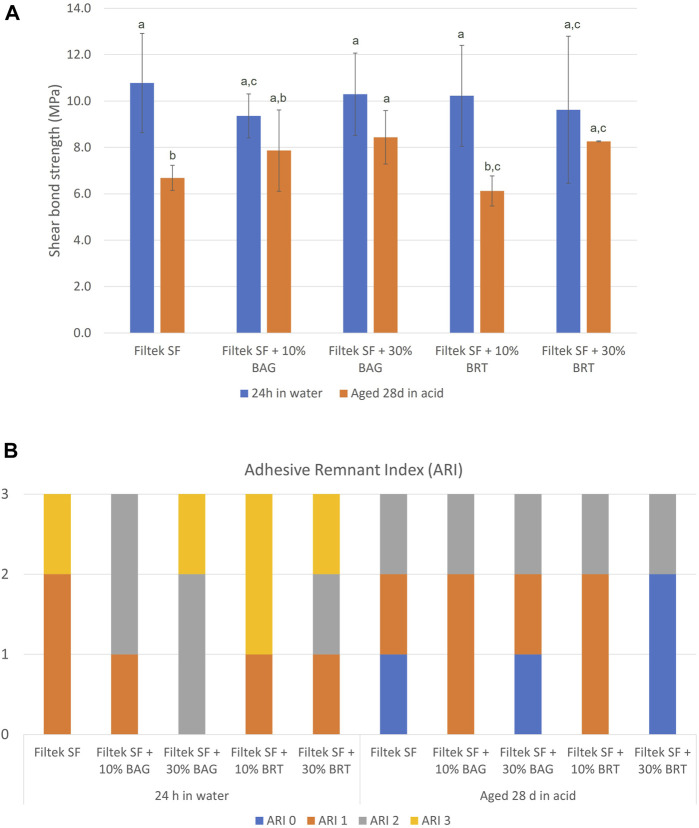
Shear testing of bioceramic micro-filler containing clear aligner attachments bonded to enamel using. **(A)** Shear bond strength. Columns labeled with different letters are statistically significantly different (*p* < 0.05). **(B)** Adhesive remnant index in different subgroups.

The incorporation of BAG and BRT into Filtek SF did not compromise the bond strength of the Flitek flowable composite. The highest bond strength was 10.8 MPa from the Filtek SF group after 24 h of storage in water (Figure 4A; Supplementary Datasheet S1). No statistically significant difference was observed between all four experimental groups and the Filtek SF group (*p* > 0.05). However, the bond strength was significantly reduced after 28 days of aging in the pH 4 demineralization solution for the Filtek SF and Filtek SF + 10% BRT groups (Figure 4A). No statistically significant difference in bond strength was detected between the 24 h in water and 28 days in acid storage conditions in the BAG and 30% BRT groups (*p* > 0.05). The 30% BAG and 30% BRT groups exhibited significantly higher shear bond strength than the Filtek SF control (*p* < 0.05).

The ARI scores are depicted in Figure 4B. After the acid challenge, there were more score “0” samples in the aging in demineralization solution groups than the 24 h in water groups. However, no significant difference in debonding patterns was observed between groups. Furthermore, no statistically significant difference was detected among all the groups regardless of the storage types (*p* > 0.05).

## 4 Discussion

The current study enabled us to investigate, for the first time, the antibiofilm and remineralization effects of bioceramic micro-fillers containing clear aligner attachments on human enamels, which brought us one step closer to establishing *in vitro* models representing clinical clear aligner therapy. The observed outcomes highlight the potential fortification achievable through the integration of bioceramic micro-fillers in clear aligner applications. While previous studies have explored the antibiofilm effects of various orthodontic cements, one major limitation was the predominant use of *S. mutans* in assessing the antimicrobial effects of orthodontic adhesives (Altmann et al., 2015; Kim et al., 2018; Liu et al., 2018). Often only a single strain (e.g., *Streptococcus Sobrinus* and *Lactobacillus casei*) was used (Imazato et al., 2003; Salehi et al., 2015). It is crucial to note that bacteria in white spot lesions and dental caries all originate from tooth surface plaque (Jakubovics and Kolenbrander, 2010). Unlike previous studies using resin disks for antibiofilm tests, our approach involved a plaque microcosm biofilm to assess the antibiofilm effects of restorative dental adhesives (Zhang et al., 2015a; Zhang et al., 2015b). Moreover, the lack of available data on the anti-plaque effectiveness of bioceramic micro-fillers containing clear aligner attachment on enamel surfaces underscores the novelty of our findings. Importantly, our minimally invasive approach preserves the biofilm around the clear aligner attachments, providing a more realistic representation of the clinical scenario.

Clinical studies have highlighted the challenge of biofilm accumulation with orthodontic appliances and emphasized the importance of managing plaque to prevent adverse effects on oral health. The present study addressed this challenge by demonstrating reduction in biofilm growth and bactericidal effects of attachments with bioceramic micro-fillers on human enamels. The value of clear aligner attachments with inherent antibiofilm properties lies in the sustained protection against plaque biofilm growth. Unlike intermittent mechanical cleaning, an attachment with intrinsic antibiofilm effects serves as a continuous source of protection. In the present study, the incorporation of bioceramic micro-fillers at a rate of 30% resulted in over 35% killing of 1-week-old plaque (Figure 2). Even at the lower incorporation percentage of 10%, there was a notable 25% reduction in 1-week-old biofilm. This aligns with a previous study showing that BRT successfully inhibited plaque biofilm formation, suppressing over 40% of biovolume in 2 weeks (Shen et al., 2016). Our findings indicated that older plaque biofilm exhibited increased resistance to antibiofilm effects compared to younger biofilm. Three-week-old plaque displayed amore mature structural development of the biofilm compared to the 1-week-old plaque. The physical barrier provided by the extracellular polymeric matrix and the presence of more persister cells both contributed to the higher resistance of mature biofilm (Xiao et al., 2012).

While both BAG and BRT groups showed comparable antibiofilm efficacy, their mechanism may differ slightly. Our study chose the well-studied bioactive glass 45S5 due to its widespread recognition as the most utilized bioactive glass with established antibacterial and remineralization potentials (Waltimo et al., 2007). The antibiofilm effect of BAG-containing attachments appears to be associated with the high surface area of BAG micro-fillers and the release of ionic components (e.g., K^+^, Na^+^, and Ca^2+^) (Waltimo et al., 2007). The exchange of network-modifier ions with H^+^ and H_3_O^+^ ions from surrounding gingival fluids results in an alkaline microenvironment with high local pH (Drago et al., 2015). Additionally, the release of PO_4_^3-^, Si^4+^, and Ca^2+^ from the BAG enhances perturbations of the membrane potential of bacteria, leading to increased osmotic pressure (Begum et al., 2016). The sudden rise in the external solutes concentration induces rapid water efflux and a pressure drop across the bacterial cell membrane, resulting in altered cell size and cell lysis as shown in the SEM (Figure 3). On the other hand, the antibiofilm effects of BRT-containing attachments may be more related to the Mg^2+^ ions (Robinson et al., 2010). Upon interaction with surrounding body fluid, Mg(OH)_2_ can formed with a pH above 11 (Shen et al., 2016). The released Si at a high pH environment may synergistically inhibit bacterial viability by acting as a surfactant to modify the cellular integrity of bacteria (Shen et al., 2016). A recent study has highlighted BRT’s ability to reinforce biological properties of a Mg–Zn bio-composite (Zhang et al., 2023). The addition of BRT to the bio-composite effectively prevented the growth and invasion of *Escherichia coli* and *Staphylococcus aureus*. This evidence supported the positive impact of BRT on flowable composite in the present study. Interestingly, there was no significant difference in the antibiofilm effects of different attachments against plaque from the two donors (*p* > 0.05). This result suggests that the source and possible differences in the species composition of biofilms from patient wearing clear aligners had no substantial impact on their susceptibility to the bioceramic micro-fillers.

Enamel demineralization is a common clinical issue during orthodontic treatment. Preserving enamel health is a priority in clinical orthodontic practice, and the innovative attachments may contribute to minimizing the risk of white spot lesions and caries (Azeem and Hamid, 2017). The present study used cross-sectional microhardness to evaluate the remineralization effects of the clear aligner attachments. The microhardness values of the lingual enamel surfaces were the highest, consistent with previously published human enamel hardness values (Galo et al., 2015). Lactic acid was used as the demineralization solution. This mimicked the biofilm acids that the orthodontic adhesive-enamel bonded areas would encounter *in vivo*, potentially affecting the resin-enamel bond strength (Langhorst et al., 2009). With the addition of bioceramic microfillers, the microhardness values significantly increased on the attachment side. When the proportion of bioceramic microfillers reached 30%, no statistically significant difference was detected between the buccal side with attachments and lingual enamel with no demineralization (*p* > 0.05). This result revealed a significant remineralization potential of BAG and BRT. Bioactive glass degrades and releases calcium and phosphate ions, acting as an external mineral ion source that accelerates remineralization (Wang et al., 2011). Apatite growth on the demineralized surfaces filled the gaps, leading to a relatively intact subsurface. Subsequent precipitation of a polycondensated silica-rich layer (Si-gel) served as a template for the formation of a calcium phosphate (Ca/P), which then crystallized into hydroxycarbonate apatite (Chen et al., 2008). The intact subsurface might be the primary reason for the higher subsurface hardness found in the experimental groups containing BAG.

A prior study demonstrated that BRT could activate the Wnt/β-catenin signalling pathway in periodontal ligament cells, promoting cementogenic/osteogenic differentiation (Zhou et al., 2013). The observed increase in microhardness may be attributed to its potential to stimulate periodontal bone formation (Shen et al., 2016), which is beneficial for restoring dental hard tissues. However, when only 10% BRT was added to the flowable composite, the microhardness in the cervical area showed no statistically significant difference with the control group (Figure 3). The lack of significance could be attributed to the crystallization of BRT in the presence of Mg^2+^, which differs from the BAG remineralization process where hydroxycarbonate apatite dominates the precipitation in the demineralized lesions.

The ability to maintain robust shear bond strength is aligned with the contemporary emphasis on attachment resilience over the course of orthodontic treatment (Weir, 2017). The shear bond strength results provided valuable insights into the performance of the developed bioceramic filler-containing clear aligner attachment. Importantly, no statistically significant difference in shear bond strength was found between the experimental groups and the control group. This suggested that the incorporation of bioceramic micro-fillers did not adversely affect the bonding efficacy (Figure 4). This finding is consistent with previous research indicating that the addition of micro-fillers can maintain bond strength without compromising shear bond properties (Guan et al., 2001; Yang et al., 2022).

The significant reduction in bond strength observed after 28 days of storage in a pH 4 demineralization solution for the Filtek SF and Filtek SF + 10% BRT groups aligns with findings from studies that highlight the susceptibility of certain adhesive systems to acidic challenges (Fan et al., 2022; Garma and Ibrahim, 2022). In contrast, the 30% BAG and 30% BRT groups exhibited significantly higher shear bond strength than the Filtek SF control under the same acidic conditions, suggesting a potential protective effect conferred by the higher concentration of bioceramic micro-fillers to enhance bond durability in acidic environments. This corresponds with previous work emphasizing the acid-resistant properties of bioactive glass in dental applications (Wang et al., 2011).

The specific reasons for the reduction in bond strength in the 10% BRT group, while not observed in the 30% BRT and BAG groups, could be influenced by various factors including the particle distribution and interaction with matrix material (Nabiyev et al., 2021). It's possible that the particle distribution in the 10% BRT group was not ideal for promoting strong adhesion, whereas the 30% BRT and BAG groups had a more favorable particle arrangement. In addition, the 10% BRT group may have exhibited less favorable chemical interactions with the resin matrix, resulting in a weaker bond compared to the 30% BRT and BAG groups.

The ARI scores provided additional insights, revealing consistent debonding patterns across all groups. While the present study did not identify significant differences in debonding patterns between groups, there were more score “0” samples in the aging in demineralization solution groups than the 24 h in water groups. This result shows that the acid environment could have affected the enamel bonding interface (Liu et al., 2018).

Within the limitation of this study, our primary focus was on establishing the short-term efficacy of the new attachments. It is indeed crucial to investigate the attachment performance over prolonged durations, ultimately contributing to improved patient outcomes. Subsequent studies may also explore other bioceramic variants including other types of BAG, building upon the results gained from this foundational investigation. Future investigations could also expand the scope by examining more types of clear aligner attachments, contributing to the continuous advancement of attachment technologies in orthodontic practice.

## 5 Conclusion

The bioceramic micro-fillers modified clear aligner attachment materials revealed significant advancements in antibiofilm and remineralization properties. The attachments demonstrated bactericidal and biofilm inhibition effects proportional to the micro-filler concentration. Moreover, the innovative materials exhibited an enhanced potential for enamel remineralization, as indicated by increased microhardness compared to control groups. Importantly, the shear bond strength remained uncompromised with the addition of micro-fillers. Overall, the incorporation of bioceramic micro-fillers represents a promising approach for enhancing oral health outcomes during clear aligner therapy.

## Data Availability

The original contributions presented in the study are included in the article/Supplementary Material, further inquiries can be directed to the corresponding author.
